# “What Is Essential Is Invisible to the Eyes”: A Short Italian Version of the Spirit at Work Scale in Healthcare

**DOI:** 10.3389/ijph.2025.1607734

**Published:** 2025-04-02

**Authors:** Laura Dal Corso, Sebastiano Rapisarda, Lucia Ronconi, Damiano Girardi, Alessandra Falco

**Affiliations:** ^1^ Department of Philosophy, Sociology, Education and Applied Psychology, University of Padua, Padua, Italy; ^2^ IT and Statistical Services, Multifunctional Pole of Psychology, University of Padua, Padua, Italy

**Keywords:** spirit at work, SAWS, workplace spirituality, healthcare, personal values, workaholism, compassion satisfaction

## Abstract

**Objectives:**

We carried out two studies to contribute to the development of a shortened Italian version of the Spirit at Work Scale (I-SAWS) in healthcare and to explore SAW’s mediating role between personal values (i.e., self-transcendence and self-enhancement) and both compassion satisfaction and workaholism.

**Methods:**

Study 1 involved 180 healthcare workers (HCWs) who completed I-SAWS. In this study, an exploratory factor analysis was performed. Study 2 involved 191 HCWs who completed the short version of I-SAWS (I-SAWS-9) and the Italian version of the Portrait Values Questionnaire, Professional Quality of Life Scale, and Dutch Work Addiction Scale. In this study, a confirmatory factor analysis and a structural equation model with observed variables were carried out.

**Results:**

Study 1 identified three factors, named higher mission, optimal functioning, and joint meaning. Study 2 confirmed a three-factor model with a second-order factor, supporting the validity and reliability of I-SAWS-9. Furthermore, results confirmed the mediating role of some SAW dimensions between self-transcendence and outcomes.

**Conclusion:**

While SAW’s association with compassion satisfaction is established, its relationship with workaholism remains ambiguous. Limitations and practical implications are discussed.

## Introduction

### Spirit at Work: Theoretical Aspects

It is evident that employees, especially healthcare workers (HCWs), are increasingly seeking more than just financial compensation from their jobs. They also explore the relationship between spirituality and their work, aiming for inspiring and meaningful roles [1, 2]. In recent years, the spirit at work (SAW) concept has gained prominence in management literature [3]. However, a universally accepted definition of SAW is still lacking. Various terms such as spirit at work, spirituality at work, spirit in the workplace, and workplace spirituality are frequently used interchangeably to describe similar dimensions [4].

As indicated by Kinjersky and Skrypnek [5], “spirit (or spirituality) at work and spirituality in the workplace also appears to be used synonymously” (p. 28). Spirit refers to being connected to oneself, others, and the universe, where all things are interconnected and purposeful. Spirituality is the energy that drives individuals towards goals beyond personal gain, representing a continuous search for meaning, a deep appreciation for life, and a personal belief system. The various terms used to refer to SAW pertain to experiences at both the organizational and individual levels. At the organizational level, workplace spirituality refers to a culture driven by mission statements, leadership, and business practices that are socially responsible and value oriented. Such a culture acknowledges employees’ contributions and fosters their personal spiritual growth and wellbeing. As an individual construct, SAW is defined as recognizing that employees possess an inner life enriched by meaningful work within a community setting. This is evident when employees can fully engage their creativity, emotions, and intellect and express various facets of themselves beyond merely performing physical or intellectual tasks [5, 6]. Despite the continued use of multiple terms to describe this phenomenon, in the present study, we use SAW to refer specifically to individual experience, which aligns with Kinjerski and Skrypnek [5].

As highlighted below, the construct’s subjectivity and multidimensionality pose challenges in its definition, which lacks a common consensus: “defining spirituality in the workplace is like capturing an angel – it’s ethereal, and beautiful, but perplexing” [7] (p. 63). Among the various possible definitions, in this study we refer to Kinjerski and Skrypnek [5], who defined SAW as “a distinct state that is characterized by physical, affective, cognitive, interpersonal, spiritual, and mystical dimensions. Most individuals describe the experience as including: a physical sensation characterized by a positive state of arousal or energy; positive affect characterized by a profound feeling of wellbeing and joy; cognitive features involving a sense of being authentic, an awareness of alignment between one’s values and beliefs and one’s work, and a belief that one is engaged in meaningful work that has a higher purpose; an interpersonal dimension characterized by a sense of connection to others and common purpose; a spiritual presence characterized by a sense of connection to something larger than self; and a mystical dimension characterized by a sense of perfection, transcendence, living in the moment, and experiences that were awe-inspiring, mysterious, or sacred” (p. 37).

Many studies have found that SAW performs a mediating role, promoting various outcomes in healthcare. For example, according to Sode and Chenji [8], the promotion of innovative work behavior is facilitated by self-transcendence and spiritual transcendence through the partial mediation of SAW. Lin and colleagues [9] reported that mindfulness promotes perceived job benefits (i.e., personal growth, positive nurse-patient relationships, recognition from family and friends, positive professional perception, and a sense of team belonging) through the mediation of SAW. Dal Corso and colleagues [10] highlighted that positive supervisor behaviors (i.e., integrity, managing emotions, considerate approach) reduce burnout through the partial mediation of SAW. In addition, De Carlo and colleagues [11] found that positive supervisor behaviors had a positive indirect effect on performance through the serial mediation of SAW and work engagement. A recent review [12] found that spirituality often reduces burnout in healthcare, though inconsistencies exist due to varied definitions and measurements. Despite this, a consideration of SAW can benefit healthcare workers’ mental health.

The healthcare sector has experienced significant changes over the years, making the work increasingly demanding. A deep connection with something greater than oneself, the search for meaning, and the sense of purpose and self-transcendence associated with SAW can significantly impact this field. Indeed, SAW in healthcare plays a central role in bridging the gap between technical efficiency and the intrinsic values of care. HCWs distinguish their role from other professions because of their genuine commitment to quality patient care and authentic care behaviors. However, despite this focus, healthcare organizations often face the challenge of balancing the demands of efficient performance with the limitations of available resources. In this context, strategies need to be developed to align technical processes with healthcare’s intangible but crucial elements, such as empathy and compassion. SAW thus fosters a sense of meaning by contributing to people’s health and strengthening community connections. This dimension offers HCWs a deeper sense of purpose by aligning their personal values with organizational goals and enhancing affective commitment and organizational citizenship behaviors [13, 14]. HCWs are particularly attuned to SAW due to the nature of their work, ethical standards, and social and personal values [15, 16]. The Schwartz Value Theory [17, 18] is the most advanced and widely accepted framework in the literature. It suggests that values are enduring beliefs about desirable end-states or behaviors and serve as guiding principles in a person’s life or social existence. Schwartz identifies ten values grouped into four dimensions: self-transcendence, self-enhancement, openness to change, and conservation. Values can be compatible or in opposition to each other (e.g., benevolence, a self-transcendent value, opposes power, a self-enhancement value). In healthcare, several studies have highlighted how the value considered most important by HCWs and both medical and nursing students is self-transcendence, to the detriment of self-enhancement [19–21]. Furthermore, similar results were also found for informal caregivers [22]. Self-transcendence values focus on the wellbeing of others and altruistic goals, encompassing principles like social justice, equality, and environmental care. These values, including universalism and benevolence, prioritize the welfare of others and discourage selfishness. In contrast, self-enhancement values, such as power and achievement, emphasize personal success, dominance, and material gain, often at the expense of others. Research highlights the theoretical and empirical association between self-transcendence and SAW, as individuals who experience self-transcendence usually seek meaning and prioritize relationships, ethics, and service to others within their work environment [8, 23–26]. Thus, a positive association between self-transcendence and SAW can be assumed. As self-enhancement is the opposite of self-transcendence, a positive association with SAW is not expected.

As previously outlined, SAW is often associated with job satisfaction [3], even in healthcare [27]. In addition to job satisfaction, compassion satisfaction is paramount in healthcare [28–31], defined as the pleasure one receives from helping others and feelings of positively contributing to the work setting, colleagues, and society [32]. SAW – specifically meaningful work and a sense of community – seems to promote HCWs’ compassion satisfaction, thus improving their professional quality of life [33]. A positive association between SAW and compassion satisfaction can therefore be assumed in line with the literature.

However, working in healthcare is a double-edged sword that can potentially positively and negatively impact an individual’s life and health. HCWs often exhibit high concern with their job, which can lead to workaholism [34–36]. Workaholism is characterized by an incessant need to work, which can adversely affect physical, mental, psychological, and social health. In the literature, many definitions of workaholism have been proposed. Spence and Robbins [37], for example, define the phenomenon as “a set of attitudes classified into three components: work involvement (limits between work and personal life), drive (internal motivation), and enjoyment of work (satisfaction obtained with work)” (p. 2). In their three identified dimensions, the authors distinguish positive and negative aspects of workaholism. The combination of these different levels within these dimensions gives rise to specific profiles of workaholics with varying degrees of functionality, ranging from those who are involved in and enjoy their work (e.g., work enthusiasts) to those who are addicted to it (e.g., work addicts). Another widely used definition in the literature is by Schaufeli and colleagues [38], who define workaholism as a tendency to work excessively and compulsively. The authors subsequently identify two dimensions: working excessively and working compulsively. Contrary to Spence and Robbins, the authors conceptualize workaholism as a negative construct, excluding pleasure and involvement in working. Once again, the possibility exists to identify specific workaholic profiles by combining the scores of the two dimensions (e.g., compulsive worker, hard worker). In the present study, we align with Schaufeli and colleagues’ definition, as it is more widely utilized by researchers who characterize workaholism as a behavioral pattern rather than an authentic work addiction [39]. Few studies have explored the relationship between SAW and workaholism, and the results are sometimes ambiguous. Although SAW is often linked to wellbeing, its relationship with workaholism is not always negative. In some instances, it tends to be positively associated with workaholism [40]. Mónico and Margaça [41] observe that SAW dimensions increase the components of workaholism in different ways.

Although it is an essentially subjective experience, the literature suggests the critical role of SAW in fostering individual and organizational wellbeing and preventing work-related stress, tangibly and concretely, as expressed by the fox’s phrase “What is essential is invisible to the eyes,” in Antoine de Saint-Exupéry’s renowned work, *The Little Prince*, which provides the title of the present paper.

### Spirit at Work: Measurement Issues

The inherent complexity of SAW makes its definition and measurement a challenging task. This challenge arises from the researchers’ endeavor to objectify an inherently subjective experience, the multidimensionality of the construct, and the overlaps created with the concept of religiosity [42]. A recent review [43] identified 18 scales for measuring workplace spirituality with individual and organizational focus, comprising vertical (i.e., connection to the transcendent, including a higher power or sacred) and horizontal aspects of spirituality (i.e., manifestation of spirituality in one’s functioning and experiences in the workplace). The Spirit at Work Scale (SAWS) by Kinjerski and Skrypnek [44, 45] focuses on individual experiences, and its dimensions include both horizontal and vertical aspects, as follows. The main reasons for using the SAWS are both its capacity to distinguish between spirituality and religiosity without referring to God, enabling individuals to ascribe their meaning to “something greater,” and to describe the individual’s experience of spirit at work clearly.

In line with the definition of SAW above, Kinjerski and Skrypnek [44, 45] developed the SAWS, which consists of 18 items grouped into four dimensions: engaging work (i.e., profound feelings of wellbeing, a belief that one is engaged in meaningful work that has a higher purpose, and an awareness of alignment between one’s values and beliefs); mystical experience (i.e., a positive state of energy or vitality, a sense of perfection, transcendence, and experiences of joy and bliss); spiritual connection (i.e., a sense of connection to something larger than self); and sense of community (i.e., feelings of connectedness to others and common purpose). Although the eigenvalue of the sense of community did not reach the conventional threshold (eigenvalue = 0.93), the authors retained it because the scree plot suggested four factors [45]. In general, SAWS shows good psychometric properties regarding reliability and test-retest reliability. SAWS also shows good convergent and discriminant validity, with positive correlations with some SAW aspects (e.g., gratitude) and work-related measures (e.g., organizational commitment) and weaker correlations with personality dimensions (e.g., conscientiousness). The author also observes negative correlations between SAW and symptoms of burnout and depression [45]. A primary merit of SAWS is its detachment from the religious dimension, often mistakenly associated with SAW. This makes it particularly suitable to be studied in organizational contexts.

Several authors have adapted the SAWS to their respective cultural contexts in the last decade. Results have frequently suggested a decrease in the number of items and factors. Specifically, Kırklıkçı [46] maintained an 18-item scale divided into three factors, while Tevichapong [47] reduced the scale from 18 to 12 items, divided into three factors. Moll [48] implemented a single-factor scale with five items. Dal Corso and colleagues [10, 11] adopted the scale for the Italian context. Results suggested maintaining the 18 items grouped in the four original factors. These findings suggest that maintaining SAWS’s original factorial structure in different cultural contexts is challenging. Currently, there is still interest in measuring SAW and workplace spirituality appropriately [43].

This paper aims to present the short Italian version of the SAWS, assessing its factorial structure, reliability, construct validity (i.e., convergent and divergent validity), and criterion validity (i.e., concurrent validity). Specifically, we aim to develop a shortened version of SAWS to make it more efficient, reduce administration time, and allow the investigation of more constructs with fewer items, ensuring the measure remains valid, reliable, concise, and free of redundancy. We carried out two studies. The first focused on evaluating the scale’s reliability and structure through exploratory factor analysis (EFA). In the second study, we aimed to confirm the stability of the factorial structure using confirmatory factor analysis (CFA) and to assess convergent, divergent, and concurrent validity by examining the correlations between the SAW’s dimensions and various theoretically relevant constructs (i.e., self-transcendence, self-enhancement, compassion satisfaction, and workaholism). Additionally, we explored the role of SAW and its dimensions as a mediating variable in the relationship between the personal values of self-transcendence and self-enhancement and both compassion satisfaction and workaholism.

## Study 1

### Methods

#### Participants and Procedure

Participants were eligible to participate if they were 18 or older. All of them worked in various healthcare organizations (e.g., hospitals). Participants were 180 HCWs living in an Italian urban area. Most participants (98.9%) indicated their age, ranging from 21 to 69, with an average of 39.7 (*SD* = 12.6); 1.1% did not. Over half of the HCWs were female (60.6%), while 38.9% were male; one missing data (0.5%). The majority held a university degree (71.7%); 20.6% had a high school diploma or professional qualification; 7.2% had a middle school diploma; one missing data (0.5%). Most participants were nurses (69.4%); 17.2% were medical doctors; 11.7% were other HCWs (e.g., physiotherapists); three missing data (1.7%). Concerning work experience, 57.8% had been with their company for one to 3 years, and 41.1% for more than 3 years; two missing data (1.1%).

Regarding participation in training courses on the humanization of assistance and care, 43.3% stated that they had participated in the last 3 years, while 55.6% said they had not attended; two missing data (1.1%). Almost half of the participants considered training on the topic quite important (34.4%); 33.9% thought it very important; 7.8% considered it not very important; and 5% not important; 18.9% did not respond. Finally, how much the role of spirituality was recognized in the working context of the participants was investigated: 53.9% stated that it is taken into little consideration; 28.3% not at all; 11.1% quite a bit; and 5% very much; three missing data (1.7%).

Participants were administered a self-report questionnaire to measure their feelings about SAW and the other study variables. A cover letter provided information regarding the study’s aim, anonymity assurance for participants, the data’s treatment, and the procedures for completing the questionnaire. Researchers were available to answer any questions participants had. They highlighted, for example, the voluntariness of their involvement, that the data collected would be used in aggregate form and for exclusive research purposes, and that the research had not been commissioned by the organization for which the participants worked. Once the participants provided their informed consent and filled out the questionnaire individually, not leaving any identifying marks, they submitted it in an enclosed box or envelope.

#### Measures

SAW was assessed using the Italian version of the SAWS (I-SAWS) [10, 11]. The scale is composed of 18 items and comprises four subscales: engaging work (“I am fulfilling my calling through my work”), mystical experience (“At moments, I experience complete joy and ecstasy at work”), spiritual connection (“I experience a connection with a greater source that has a positive effect on my work”), and sense of community (“I experience a real sense of trust and personal connection with my coworkers”). The response scale ranged from 1 (completely untrue) to 6 (completely true).

Socio-demographic information, the frequency with which participants participate in training courses on humanization, and the importance and recognition of spirituality in their organization were all assessed.

#### Data Analysis

We conducted our analyses using R software version 4.4.0. Exploratory Factor Analysis (EFA) was performed using the principal axis extraction and Promax rotation methods to define the I-SAWS structure. Kaiser–Meyer–Olkin (KMO) and Bartlett’s test of sphericity was performed to assess the suitability of the scale for EFA. A KMO value >0.80 or a *p*-value of Bartlett’s test <0.05 indicates adequate sample size and suitability of variables for factor analysis. We examined eigenvalues over 1 and the scree plot to define the number of factors. We fixed a cut-off point of >0.60 for factor loadings to have a more manageable tool and selected the three most representative items for each dimension.

### Results

The KMO value was 0.82, and Bartlett’s test of sphericity was significant – χ^2^ (153) = 1,366.28, *p* < 0.001. The data were ideal for factor analysis. There were three eigenvalues over 1 (5.03, 1.46, 1.06). The scree plot examination confirmed the selection of three factors (see Supplementary Material S1). Table 1 shows factor loadings of 18 items of I-SAWS after rotation. We retained the nine items with factor loadings over 0.60 (i.e., Item 11, Item 15, Item 10, Item 7, Item 5, Item 8, Item 17, Item 3, Item 18).

**TABLE 1:** Factor loadings of Italian version of the Spirit at Work Scale and Cronbach’s alpha (Italy, 2017–2024).

Items	Factor 1	Factor 2	Factor 3
	Higher mission	Optimal functioning	Joint meaning
Item 11 (EW)	**0.95**	−0.05	−0.11
Item 15 (SpC)	**0.91**	−0.21	0.06
Item 10 (SpC)	**0.87**	−0.25	0.03
Item 9 (EW)	0.55	0.26	−0.15
Item 6 (SpC)	0.54	−0.05	0.14
Item 14 (EW)	0.39	0.30	−0.04
Item 2 (ME)	0.36	0.00	−0.06
Item 7 (EW)	−0.17	**0.94**	−0.09
Item 5 (ME)	−0.26	**0.89**	0.04
Item 8 (ME)	0.07	**0.73**	−0.05
Item 4 (EW)	−0.04	0.60	0.11
Item 16 (ME)	0.22	0.33	0.21
Item 12 (ME)	0.19	0.30	−0.06
Item 17 (SoC)	−0.04	−0.03	**0.87**
Item 3 (SoC)	−0.07	−0.23	**0.74**
Item 18 (EW)	−0.01	0.19	**0.61**
Item 13 (SoC)	0.04	0.09	0.51
Item 1 (EW)	0.14	0.11	0.27
Cronbach’s alpha	0.87	0.82	0.74

Note. Factor loadings over 0.60 are reported in bold. The original factor the items load on is indicated in brackets. EW, engaging work; SpC, spiritual connection; ME, mystical experience; SoC, sense of community.

The nine items initially grouped under the engaging work dimension were distributed into the other three factors (i.e., spiritual connection, mystical experience, and sense of community), respectively. Factor 1, resulting from combining the original factors of engaging work and spiritual connection and named *higher mission*, is defined as a sense of connection to something greater than oneself, which inspires and guides the individual in their work. It also refers to the perception of having a mission in life, a greater purpose, which the work helps to pursue. Higher mission explained 18% of the variance and comprises Item 11, Item 15, and Item 10. Factor 2, named *optimal functioning*, resulted from combining engaging work and mystical experience, explained 16% of the variance and comprises Item 7, Item 5, and Item 8. This dimension is characterized by a positive state of energy or vitality, experiences of joy and bliss, and a strong passion for one’s work. Finally, combining the engaging work and sense of community dimensions led to Factor 3, named *joint meaning*. It explained 12% of the variance and comprises Item 17, Item 3, and Item 18. Joint meaning is characterized by feelings of connectedness and trust with colleagues, a sense of belonging to a community, and the perception of being in just the right place.

## Study 2

### Methods

#### Participants and Procedure

Participants were eligible to participate if they were 18 or older. All of them worked in various healthcare organizations (e.g., hospitals). Participants were 191 HCWs living in an Italian urban area. The majority of participants (97.9%) indicated their age, which ranged from 21 to 69, with an average of 39.7 (*SD* = 10.30); 2.1% did not. Most participants were female (75.4%), while 24.6% were male. Over half of the HCWs held a university degree (62.8%); 37.2% had a high school diploma or professional qualification. Most participants were nurses (71.2%); 21.5% were medical doctors; 5.5% were other HCWs (e.g., midwives); 1% were healthcare assistants; one missing date (0.5%). Concerning work experience, 49.2% had been with their current company for one to three years; 44.5% for more than three years; 12 missing data (6.3%).

Regarding participation in training courses on the humanization of assistance and care, 48.7% stated that they had participated in the last three years, while 50.8% said they had not attended; one missing data (0.5%). Most HCWs considered training on the topic quite important (36.7%) or very important (36.1%); 5.2% considered it not very important; and 1.1% not at all important; 20.9% did not respond. Finally, how much the role of spirituality was recognized in the working context of the participants was investigated: 57.6% stated that it is taken into little consideration; 25.1% not at all; 14.7% quite a bit; and 1.6% very much; two missing data (1%).

General procedures were identical to those adopted in Study 1.

#### Measures

In the present study, SAW was assessed with the nine items demonstrating factor loading over 0.60 in Study 1. Our final version (named Italian Spirit at Work Scale-9; I-SAWS-9; see Supplementary Appendix SA1) resulted in a three-factor scale with three items each: higher mission (e.g., “I have a sense of personal mission in life, which my work helps me to fulfill”), optimal functioning (e.g., “I am passionate about my work”), and joint meaning (e.g., “I feel like I am part of “a community” at work”). The response scale ranged from 1 (completely untrue) to 6 (completely true). Cronbach’s alpha for the three sub-scales reported above is 0.84, 0.74, and 0.71, respectively. Cronbach’s alpha for the overall scale is 0.79.

Self-transcendence and self-enhancement were assessed with the Italian version of the Portrait Values Questionnaire (PVQ40) by Schwartz [49]. Self-transcendence is composed of ten items and comprises benevolence (e.g., “It’s very important to him/her to help the people around him/her. He/She wants to care for their wellbeing”) and universalism (e.g., “He/She thinks it is important that every person in the world be treated equally. He/She believes everyone should have equal opportunities in life”). Self-enhancement is composed of seven items and comprises achievement (e.g., “He/She thinks it is important to be ambitious. He/She wants to show how capable he/she is”) and power (e.g., “He/She always wants to be the one who makes the decisions. He/She likes to be the leader”). The response scale ranged from 1 (not at all similar to me) to 6 (very similar to me). The Cronbach’s alpha for the scales was 0.83 and 0.85, respectively.

Compassion satisfaction was assessed with the Italian version of the Professional Quality of Life Scale (ProQOL) [50]. The compassion satisfaction subscale comprises ten items (e.g., “I get satisfaction from being able to help people”). The response scale ranged from 0 (never) to 5 (very often). The Cronbach’s alpha for the scale was 0.82.

Workaholism was evaluated using the Italian adaptation of the Dutch Work Addiction Scale (DUWAS) [38, 51, 52]. The scale is composed of ten items and comprises the two dimensions of workaholism: working excessively (e.g., “I find myself continuing to work after my co-workers have called it quits”) and working compulsively (e.g., “I feel that there’s something inside me that drives me to work hard”). The response scale ranged from 1 (strongly disagree) to 6 (strongly agree). The Cronbach’s alpha for the scale was 0.81.

The same socio-demographic information from the previous study was collected at the end of the questionnaire.

#### Data Analysis

The psychometric properties of the I-SAWS-9 were evaluated through a series of Confirmatory Factor Analyses (CFA). We conducted three CFAs (i.e., one-factor model, three-factor model, second-order model with three first-order factors), using the robust maximum likelihood (MLM) method. To assess the adequacy of the model, we used the χ^2^ test. A model shows a good fit to data if χ^2^ is nonsignificant. However, because the χ^2^ is affected by sample size, we considered additional fit indices [53]: the comparative fit index (CFI) and the nonnormed fit index (NNFI), both associated with good fit if values are ≥0.95; the root-mean-square error of approximation (RMSEA) and the standardized root-mean-square residual (SRMR), both associated with good fit if values are ≤0.08 and ≤0.10, respectively. We then calculated the composite reliability (CR) and the average variance extracted (AVE) indices, whose values ≥0.70 and ≥0.50, respectively, are considered satisfactory [54, 55]. The Chi-square difference (Δχ^2^) test was used to compare the fit of competing nested models [56]. Pearson correlations were analyzed to verify convergent, divergent, and concurrent validity.

Finally, to test the hypothesized indirect effects of self-transcendence and self-enhancement on compassion satisfaction and workaholism through the three dimensions of SAW (i.e., parallel mediation), a structural equation model with observed variables (i.e., path analysis) was estimated. The structural paths were freely estimated to test both direct and indirect effects simultaneously. The maximum likelihood (ML) and Bootstrap methods at 95% confidence intervals were used. A statistically significant mediation is supported if a confidence interval does not contain zero [57]. Statistical analyses were carried out using R software version 4.4.0.

### Results

**FIGURE 1:**
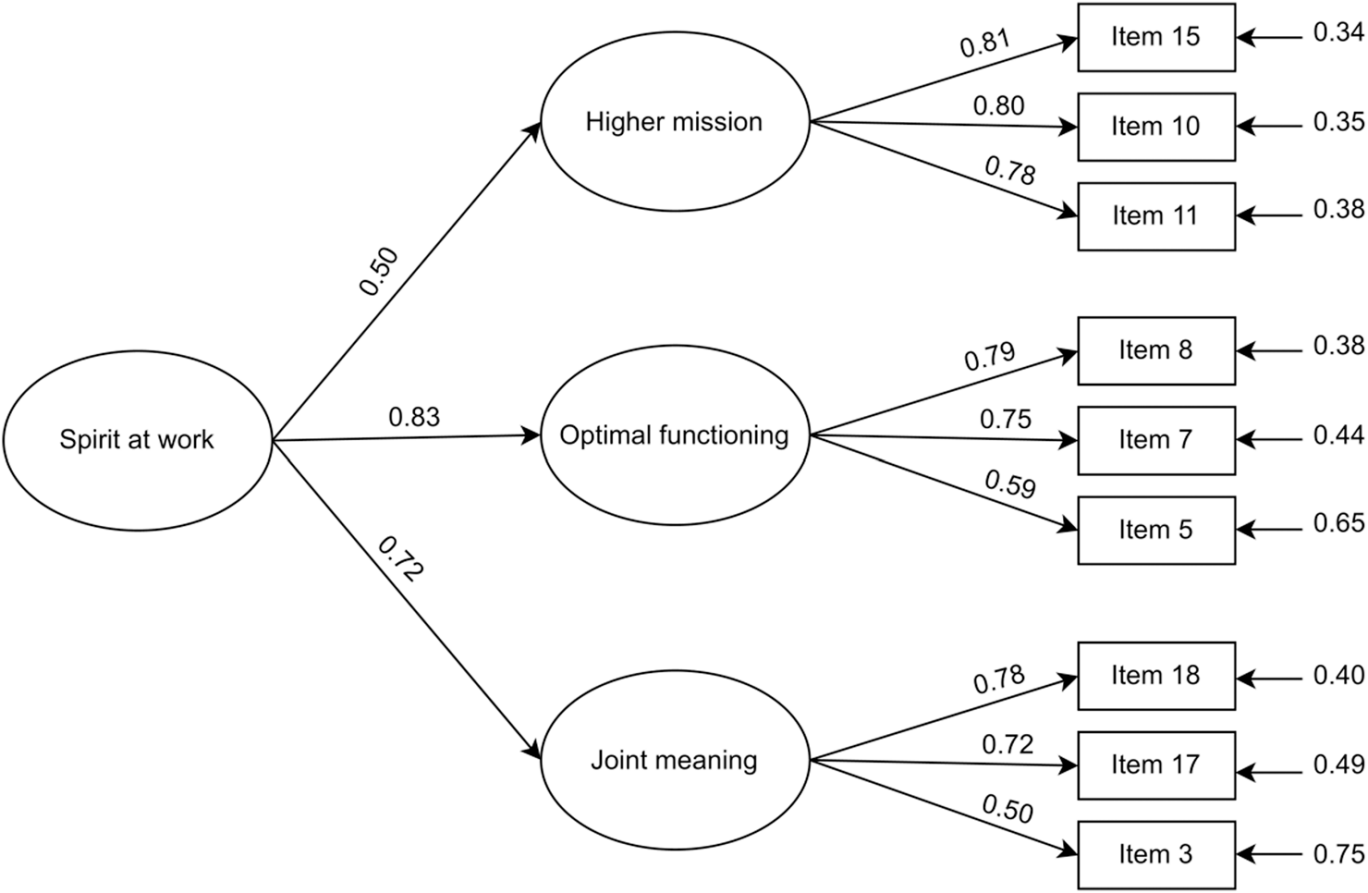
Factor loadings of the second-order model (Italy, 2017–2024). Note. The standardized factor loadings are reported.

Table 2 shows fit indexes for tested models. The one-factor model showed a bad fit to the data – χ^2^ (27) = 200.35, *p* < 0.001; CFI = 0.64; NNFI = 0.52; RMSEA = 0.18; SRMR = 0.15. In contrast, both the three-factor model and the second-order model with three first-order factors showed a good fit to the data – χ^2^ (24) = 33.28, *p* > 0.05; CFI = 0.98; NNFI = 0.96; RMSEA = 0.05; SRMR = 0.05. According to the Δχ^2^ test (Δχ^2^ (3) = 167.06, *p* < 0.001), results indicated a structure with three factors. In addition, the three first-order factors loaded strongly onto the higher-order factor in the second-order model (Figure 1).

**TABLE 2:** Fit indexes of tested models (Italy, 2017–2024).

Model	χ^2^	*df*	CFI	NNFI	RMSEA	SRMR	Model comparison		
								Δχ^2^	Δ*df*
1. One-factor model	200.35***	27	0.64	0.52	0.18	0.15			
2. Three-factor model	33.28	24	0.98	0.96	0.05	0.05	2 vs. 1	167.06***	3
3. Second-order model	33.28	24	0.98	0.96	0.05	0.05	3 vs. 1	167.06***	3

*Note*. χ^2^, chi-square; df, degrees of freedom; CFI, comparative fit index; NNFI, nonnormed fit index; RMSEA, root-mean-square error of approximation; SRMR, standardized root-mean-square residual; Δχ^2^, chi-square difference; Δdf, degrees of freedom difference. *p < 0.05. **p < 0.01. ***p < 0.001.

All the standardized coefficients were significant at the 0.05 level. Furthermore, CR and AVE reach satisfying values for all factors (i.e., higher mission – CR = 0.84, AVE = 0.64; optimal functioning – CR = 0.75, AVE = 0.51; joint meaning – CR = 0.71, AVE = 0.50). Therefore, we considered the measurement model validity appropriate.

Table 3 shows the psychometric properties of each variable in the mediation model. The skewness and kurtosis coefficients ranged from −1 to 1. We verified the internal reliability of each scale by calculating Cronbach’s alpha coefficient, which showed a high degree of internal reliability for all scales. Moreover, Table 3 presents the correlations among the measured psychological dimensions. Convergent, divergent, and concurrent validity were examined through bivariate correlations. The positive and significant correlations between the three dimensions of SAW and self-transcendence indicate appropriate convergent validity. Only joint meaning showed a low correlation with self-enhancement (*r* = 0.15; *p* < 0.05), indicating appropriate divergent validity; the significant correlations between the three dimensions of SAW and compassion satisfaction, and the correlation between the dimension optimal functioning of SAW and workaholism, indicate appropriate concurrent validity.

**TABLE 3:** Psychometric properties and correlations among the measured psychological dimensions (Italy, 2017–2024).

Variables	*M* (*SD*)	Skewness	Kurtosis	1	2	3	4	5	6	7	8
1. Higher mission	3.90 (1.30)	−0.43	−0.65	(0.84)							
2. Optimal functioning	4.59 (0.90)	−0.51	−0.09	0.32***	(0.74)						
3. Joint meaning	3.99 (1.02)	−0.39	−0.09	0.26***	0.41***	(0.71)					
4. Spirit at work	4.16 (0.80)	−0.26	−0.29	0.77***	0.73***	0.72***	(0.79)				
5. Self-transcendence	4.82 (0.65)	−0.70	0.42	0.31***	0.23**	0.19**	0.33***	(0.83)			
6. Self-enhancement	3.31 (0.96)	−0.13	−0.66	−0.01	0.11	0.15*	0.10	0.05	(0.85)		
7. Compassion satisfaction	3.56 (0.67)	−0.23	−0.07	0.35***	0.51***	0.33***	0.52***	0.35***	0.21**	(0.82)	
8. Workaholism	3.70 (0.83)	0.31	−0.32	0.10	0.23**	−0.05	0.12	0.24**	0.17*	0.17*	(0.81)

*Note*. M and SD are used to represent mean and standard deviation, respectively. Cronbach’s alphas are shown in brackets. *p < 0.05. **p < 0.01. ***p < 0.001.

**FIGURE 2:**
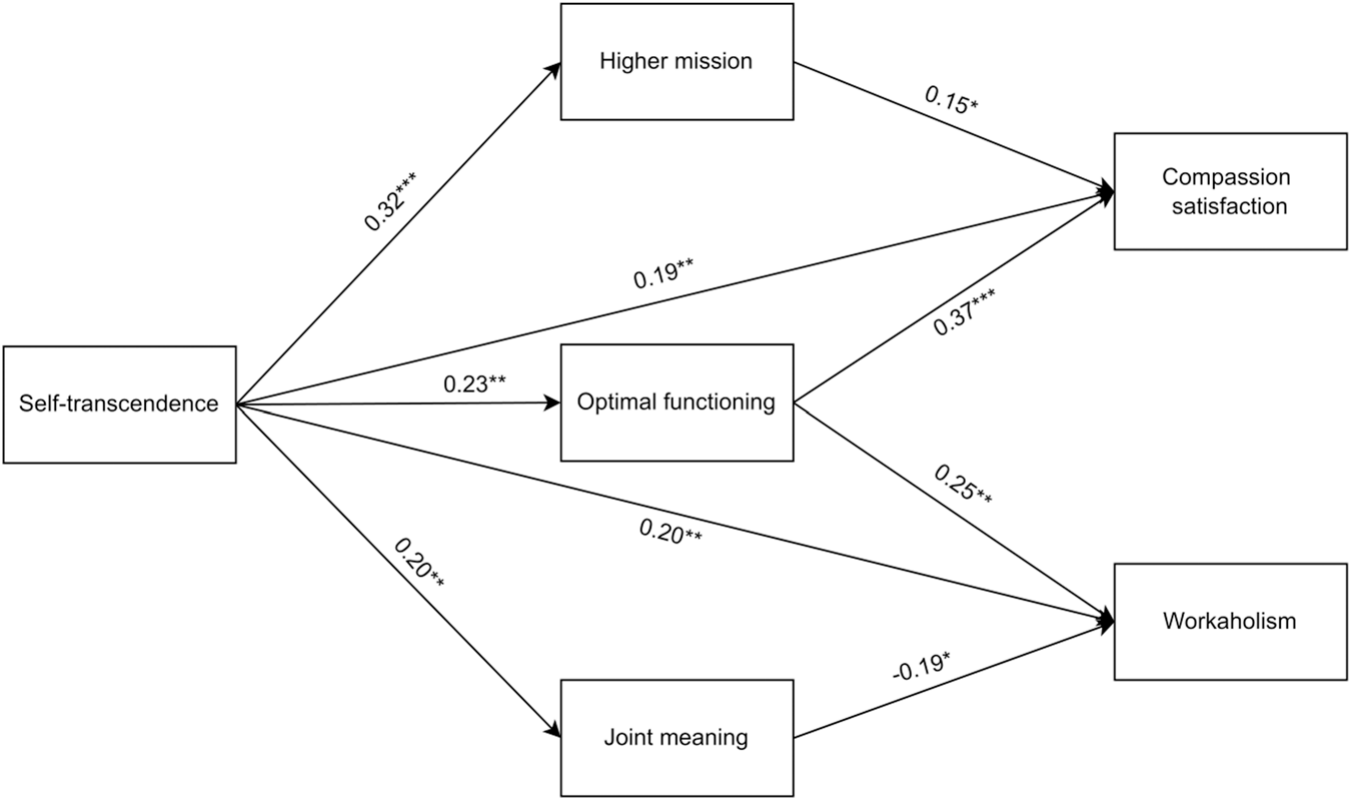
The path analysis model (Italy, 2017–2024). Note. In the path analysis model, only significant relationships are reported. *p < 0.05. **p < 0.01. ***p < 0.001.

Finally, a path analysis model was estimated to test the hypothesized relationships between self-transcendence, self-enhancement, the three dimensions of SAW, compassion satisfaction, and workaholism. After controlling for the participation in training courses on the humanization of assistance, self-enhancement was associated only with compassion satisfaction (β = 0.15, *p* < 0.05) and workaholism (β = 0.17, *p* < 0.05), while the relationships with the dimensions of SAW were not significant. We excluded self-enhancement from the model to explore the role of SAW dimensions as mediators and achieve a more parsimonious solution. In this second model (Figure 2), self-transcendence was positively associated with higher mission (β = 0.32, *p* < 0.001), optimal functioning (β = 0.23, *p* < 0.01), and joint meaning (β = 0.20, *p* < 0.01). Furthermore, self-transcendence was positively associated with compassion satisfaction (β = 0.19, *p* < 0.01) and workaholism (β = 0.20, *p* < 0.01), controlling for the effect of the three SAW dimensions. Moreover, higher mission and optimal functioning were positively associated with compassion satisfaction (β = 0.15, *p* < 0.05; β = 0.37, *p* < 0.001), whereas joint meaning was not. Interestingly, optimal functioning was positively associated with workaholism (β = 0.25, *p* < 0.01), but joint meaning was negatively associated with it (β = −0.19, *p* < 0.01), whereas higher mission was not.

We further verify the mediating role of the three dimensions of SAW between self-transcendence and both outcomes (i.e., compassion satisfaction, workaholism). When Bootstrap = 5,000, the indirect effect of self-transcendence on compassion satisfaction through higher mission (95% CI = 0.002, 0.102) and optimal functioning (95% CI = 0.027, 0.154) was significant. Moreover, the indirect effect of self-transcendence on workaholism through optimal functioning (95% CI = 0.012, 0.115) and joint meaning (95% CI = −0.089, −0.004) was significant. The 95% CI for the indirect effect of self-transcendence on compassion satisfaction through joint meaning and on workaholism through higher mission contained zero, indicating both indirect effects are nonsignificant (Table 4).

**TABLE 4:** Bootstrapping indirect effects (Italy, 2017–2024).

Indirect effect	Standardized parameter	Bootstrap 5000 times 95% CI	
		CI lower	CI upper
Self-transcendence→Higher mission→Compassion satisfaction	**0.047**	**0.002**	**0.102**
Self-transcendence→Optimal functioning→Compassion satisfaction	**0.086**	**0.027**	**0.154**
Self-transcendence→Joint meaning→Compassion satisfaction	0.021	−0.014	0.056
Self-transcendence→Higher mission→Workaholism	0.005	−0.052	0.059
Self-transcendence→Optimal functioning→Workaholism	**0.058**	**0.012**	**0.115**
Self-transcendence→Joint meaning→Workaholism	**−0.038**	**−0.089**	**−0.004**

Note. Significant indirect effects are reported in bold. CI is used to represent confidence intervals.

## Discussion

In this research, we aimed to verify the psychometric properties of the I-SAWS in Italian healthcare and proposed a new short version. The data from these two studies supported the validity and reliability of the I-SAWS-9 as a measure of SAW. Results showed a second-order model with three first-order factors and three items each. Results also confirmed the partial mediating role of the three SAW dimensions: self-transcendent showed a positive indirect effect on compassion satisfaction through higher mission and optimal functioning. Interestingly, self-transcendent showed a positive indirect effect on workaholism through optimal functioning but a negative indirect effect on it through joint meaning. No mediation effects of SAW dimensions appeared in the relationship between self-enhancement and outcomes. According to Schwartz [17, 18], self-enhancement values, characterized by power and achievement, conflict with self-transcendence values, characterized by universalism and benevolence. Given that self-transcendence values are those most associated with SAW [8, 24], the results of the present study align with the literature. While the relationship between SAW and compassion satisfaction—and in general with job satisfaction—aligns with existing literature [3, 33, 58], the relationship between the dimensions of SAW and workaholism (i.e., a positive relationship with optimal functioning and a negative relationship with joint meaning) appears ambiguous. Mydin and colleagues [40] showed a positive association between SAW and workaholism, while Mónico and Margaça [41] found positive associations between SAW and some specific workaholic profiles. Our findings partly align with the literature, as we find a positive relationship between optimal functioning and workaholism and a negative relationship between joint meaning and workaholism. The divergent findings across studies could be attributed to the varying conceptualizations and measurement tools employed in assessing workaholism. This result highlights a dark side of SAW, as its dimension focused on joy, energy, passion to perform a task, and a profound sense of purpose that may result in a strong drive to work, potentially to the detriment of other domains of life (e.g., family), contributing to the development of workaholism. However, it is important to note that the extant literature on this subject is limited, and further research is necessary to better understand the relationship between these two constructs.

Nonetheless, this research has some limitations. While the I-SAWS-9 demonstrated satisfactory psychometric properties, its three-factor structure differed from the original four-factor model proposed by Kinjerski and Skrypnek [44, 45]. This discrepancy may be attributed to the reduced number of items and/or the distinct cultural context, as suggested by other researchers [46, 47]. However, the presence of one of the four dimensions did not fail; instead, the three selected items with a factor loading greater than 0.60 of the original factor “Engaging work” are equally distributed in the three factors derived from EFA. Consequently, although the new scale has a trifactorial structure, the nine items align with Kinjerski and Skrypnek’s theorizing and successfully capture the main aspects of SAW. Moreover, the majority of HCWs in this research were nurses. However, this professional figure is also most represented in the Italian healthcare sector. Study 2 presents the limits of a cross-sectional design. Specifically, since we are not dealing with longitudinal data, it is not possible to track the evolution of variables and establish causal inferences. Additionally, the path model involves using observed variables, which can occasionally compromise the measurement accuracy of complex constructs and increase the risk of measurement error.

Future research should employ a longitudinal design to investigate the direction of the associations between personal values, SAW, compassion satisfaction, and workaholism. The study could benefit from a more balanced number of participants based on healthcare professions and gender, including considering different professional groups and cultural contexts. In addition, it may be valuable to consider the patient’s perspective (e.g., patient satisfaction) [13]. Furthermore, it is necessary to explore the relationship between SAW and workaholism to better understand the subtle boundary between SAW’s role as a protective factor against workaholism and its consequences (e.g., inflammatory response, presenteeism) [35, 59] and as a risk factor. Also, exploring the relationship between SAW and workaholism, considering other relevant dimensions (e.g., irrational beliefs at work, happiness, organizational citizenship behavior) [60–62], would be helpful. Additionally, investigating the role of SAW, also through case studies [63], is proposed to help design psychological interventions to prevent, mitigate, and limit the negative consequences of workaholism and promote the quality of professional life.

The present study’s practical implications are notable. The proposal of a short measurement scale for assessing SAW may significantly impact organizations because it allows faster and more efficient data collection. Therefore, the I-SAWS-9 reduces the time required to complete surveys in organizational contexts, makes it easier to include SAW in surveys on employees’ quality of life at work, and results in fewer missing data and a higher response rate. A short measure could facilitate the implementation of early interventions (e.g., psychological support or professional development programs) and enable continuous assessment of organizational wellbeing. Having practical and agile measurement tools is critical in public health, where employee wellbeing is crucial.

The second study’s findings indicate that aspects of SAW related to the feeling of connection to something greater than oneself, the perception of having a greater purpose (i.e., higher mission), a positive state of energy, experiences of joy and bliss, and passion for work (i.e., optimal functioning), can foster compassion satisfaction in healthcare, in which a sense of helping others plays a central role. In addition, fostering aspects of SAW associated with feelings of connection and trust with colleagues and a sense of community (i.e., joint meaning) may serve as a protective factor against workaholism. Integrating spirit at work into company policies can contribute to a more balanced and fulfilling work context.
