# Supplementary material for: “What Is Essential Is Invisible to the Eyes”: A Short Italian Version of the Spirit at Work Scale in Healthcare
Source: Int J Public Health. 2025 Apr 2;70:1607734. doi: 10.3389/ijph.2025.1607734 (PMC11999818; doi:10.3389/ijph.2025.1607734)
Supplement: Supplementary file 1 [file DataSheet2.pdf]

Supplementary Appendix 1

Italian Spirit at Work Scale-9 (I-SAWS-9)

We ask you to read each of the following statements relating to your perception of yourself and answer the question: “How true is this for you?”. Choose the appropriate value on the response scale provided, where “1” means the statement is completely untrue to you and “6” means it is completely true.

Le chiediamo di leggere ognuna delle seguenti affermazioni relative alla percezione che Lei ha di se stessa/o e di rispondere alla domanda: “Quanto vero è per Lei?”. Scelga il valore appropriato sulla scala di risposta fornita, dove “1” significa che l’affermazione è fortemente falsa per Lei e “6” indica che è fortemente vera.

Completely untrue *Fortemente falso per me*	Mostly untrue *Abbastanza falso per me*	A little bit untrue *Moderatamente falso per me*	A little bit true *Moderatamente vero per me*	Mostly true *Abbastanza vero per me*	Completely true *Fortemente vero per me*
1	2	3	4	5	6

Final version of the I-SAWS-9
1 (10). My spiritual beliefs play an important role in everyday decisions that I make at work. *Le mie credenze spirituali svolgono un ruolo importante nelle decisioni che ogni giorno devo prendere al lavoro.*
2 (11). I have a sense of personal mission in life, which my work helps me to fulfill. *Sento di avere una missione nella vita che il mio lavoro mi aiuta a perseguire.*
3 (15). I receive inspiration or guidance from a Higher Power about my work. *Credere in “qualcosa di più grande” mi ispira e mi guida nel lavoro.*
4 (5). At moments, I experience complete joy and ecstasy at work. *In certi momenti, al lavoro, provo una grande gioia.*
5 (7). I am passionate about my work. *Il mio lavoro mi appassiona.*
6 (8). At times, I experience an energy or vitality at work that is difficult to describe. *A volte, al lavoro, sento di avere una tale energia o vitalità che mi è difficile descrivere.*
7 (3). I experience a real sense of trust and personal connection with my coworkers. *Provo un vero senso di fiducia e di legame con colleghe/i.*
8 (17). I feel like I am part of “a community” at work. *Al lavoro, mi sento parte di “una comunità”.*
9 (18). At the moment, I am right where I want to be at work. *In questo momento, al lavoro, sento di essere proprio al posto giusto.*

Note. Higher mission = items 1, 2, 3. Optimal functioning = items 4, 5, 6. Joint meaning = items 7, 8, 9. Spirit at work = items 1 – 9. The position of the item in the original version of the Spirit at Work Scale is indicated in brackets.

This scale may be freely copied as long as (a) authors are credited, (b) no changes are made, and (c) it is not sold. Anyone interested in using the scale should notify the corresponding author.
