# Supplementary figures and images for: “What Is Essential Is Invisible to the Eyes”: A Short Italian Version of the Spirit at Work Scale in Healthcare
Source: Int J Public Health. 2025 Apr 2;70:1607734. doi: 10.3389/ijph.2025.1607734 (PMC11999818; doi:10.3389/ijph.2025.1607734)

## Supplementary Material 1

Scree Plot (Italy, 2017-2024)

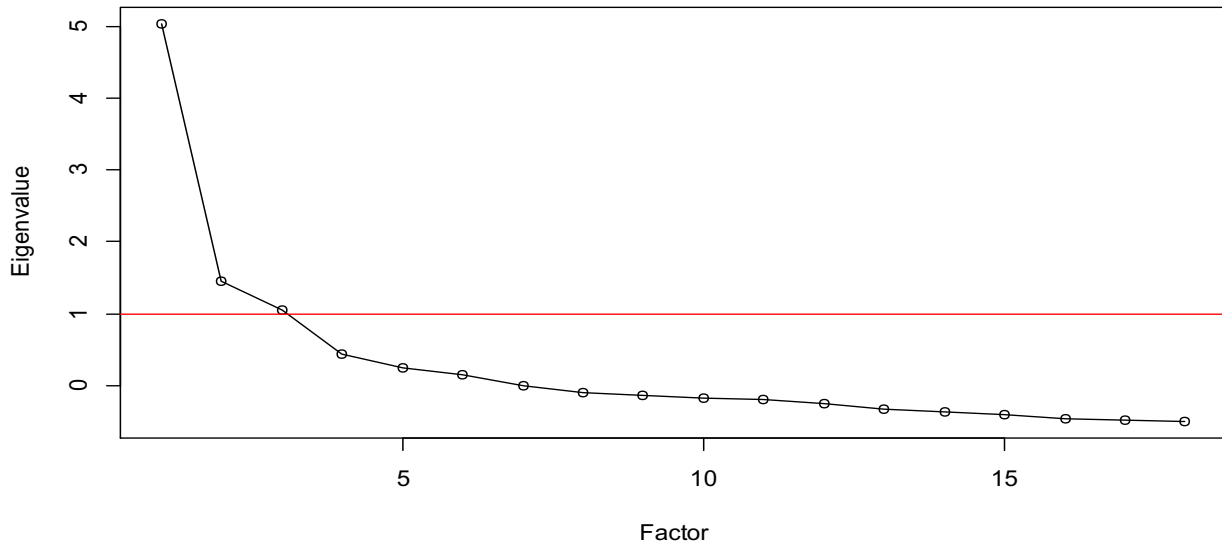

Supplement: Supplementary file 2 [file DataSheet1.pdf]
